# North Atlantic Migratory Bird Flyways Provide Routes for Intercontinental Movement of Avian Influenza Viruses

**DOI:** 10.1371/journal.pone.0092075

**Published:** 2014-03-19

**Authors:** Robert J. Dusek, Gunnar T. Hallgrimsson, Hon S. Ip, Jón E. Jónsson, Srinand Sreevatsan, Sean W. Nashold, Joshua L. TeSlaa, Shinichiro Enomoto, Rebecca A. Halpin, Xudong Lin, Nadia Fedorova, Timothy B. Stockwell, Vivien G. Dugan, David E. Wentworth, Jeffrey S. Hall

**Affiliations:** 1 National Wildlife Health Center, United States Geological Survey, Madison, Wisconsin, United States of America; 2 Southwest Iceland Nature Research Institute, Sandgerði, Iceland; 3 Snæfellsnes Research Centre, University of Iceland, Stykkishólmur, Iceland; 4 Veterinary and Biomedical Sciences Department, College of Veterinary Medicine, University of Minnesota, Saint Paul, Minnesota, United States of America; 5 J. Craig Venter Institute, Rockville, Maryland, United States of America; Duke-NUS Gradute Medical School, Singapore

## Abstract

Avian influenza virus (AIV) in wild birds has been of increasing interest over the last decade due to the emergence of AIVs that cause significant disease and mortality in both poultry and humans. While research clearly demonstrates that AIVs can move across the Pacific or Atlantic Ocean, there has been no data to support the mechanism of how this occurs. In spring and autumn of 2010 and autumn of 2011 we obtained cloacal swab samples from 1078 waterfowl, gulls, and shorebirds of various species in southwest and west Iceland and tested them for AIV. From these, we isolated and fully sequenced the genomes of 29 AIVs from wild caught gulls (Charadriiformes) and waterfowl (Anseriformes) in Iceland. We detected viruses that were entirely (8 of 8 genomic segments) of American lineage, viruses that were entirely of Eurasian lineage, and viruses with mixed American-Eurasian lineage. Prior to this work only 2 AIVs had been reported from wild birds in Iceland and only the sequence from one segment was available in GenBank. This is the first report of finding AIVs of entirely American lineage and Eurasian lineage, as well as reassortant viruses, together in the same geographic location. Our study demonstrates the importance of the North Atlantic as a corridor for the movement of AIVs between Europe and North America.

## Introduction

Avian influenza viruses (AIV) are commonly found in wild waterfowl, shorebirds and gulls around the world. While these infections do not cause significant disease among wild birds, there is some evidence of subclinical signs of low pathogenic (LP) AIV infection in free living waterfowl [1], [2]. Historically, highly pathogenic (HP) AIVs have rarely been seen in wild birds, but since 1997 when HPAIV H5N1 began circulating among domestic waterfowl and terrestrial birds primarily in southeast Asia, mortality events affecting wild birds due to this disease have been extensively documented [3], [4]. This raised the question of whether wild migratory birds facilitated the spread of HPAIV H5N1 throughout Asia, Africa and Europe and whether they will be the mechanism for introduction of this virus, or future HPAIV, into the Americas [5].

AIVs are segmented negative sense RNA viruses consisting of 8 genomic RNA segments, comprising 6 internal protein coding segments (polymerase basic 2 (PB2), polymerase basic 1 (PB1), polymerase acidic (PA), nucleoprotein (NP), matrix (M), and non-structural (NS)) and 2 segments encoding surface glycoproteins (hemagglutinin (HA) and neuraminidase (NA)). Of these 8 segments, the HA, NA, and NS segments contribute the most to the variability of AIV genomes [6]. The HA and NA are genetically very diverse segments with 18 (H1–H18) and 11 (N1–N11) different subtypes reported and allowing for classification of AIV into HA-NA subtypes [7]–[9]. The NS segment is separable into 2 distinct alleles (A and B) both of which are regularly seen in analysis of wild bird AIV [10], [11]. The remaining 5 internal protein coding segments are highly conserved with the extent of diversity in these segments less than that found within an HA or NA subtype [12].

Each segment is subject to point mutations (genetic drift) within the segment and entire segments can be replaced (genetic shift) when birds are co-infected with more than one AIV simultaneously [12], [13]. Within each of these segments clear genetic differences have evolved between the Eurasian and American viruses, including the 2 NS alleles and most HA and NA subtypes [12], [14], [15]. With increased availability of genetic data, American AIV segments are more frequently being detected in Eurasia and Eurasian AIV segments are more frequently being detected in the Americas [12], [16]–[20]. While there is limited information on the directionality of AIV gene flow, Eurasian lineage AIV segments have been introduced into North American viruses to become the dominant lineage within North America, and an American lineage segment has become established in wild birds in Australia [14], [21], [22]. In addition to the Eurasian and American lineages, a distinct gene pool of gull influenza viruses was first proposed in 1988 [23]. Recent phylogenetic analysis has confirmed the presence of a gull lineage or clade in each of the internal segments although the PB2, PB1, and PA gull clades are not as clearly defined as the NP, M, and NS gull clades [19].

Wild waterfowl, shorebirds, and gulls have extensive migrations that cross the Pacific and Atlantic oceans. This primarily occurs as birds move between breeding and non-breeding grounds in the East Atlantic, East Asian, and the East Pacific Flyways [24]. These flyways provide opportunities for migratory birds to introduce Eurasian AIV lineages into North America and vice versa.

Much of the research into movements of AIVs between Europe, Asia, and Africa and the Americas has been in Alaska due to the proximity of North America and Asia in this region and the extensive intercontinental bird migrations that occur there. AIVs containing Eurasian lineage segments have been recovered from birds in Alaska including nearly complete Eurasian AIV genomes [17], [19]. In comparison to the North Pacific, the North Atlantic region, primarily Iceland and Greenland, has been largely unstudied. In one study in the Atlantic provinces of Canada, a nearly complete Eurasian AIV genome was detected in a mallard (*Anas platyrhynchos*), with 7 segments of the AIV genome belonging to the Eurasian lineage, and 5 other birds had AIV with 4 to 5 segments of Eurasian lineage [16]. To date, there have been no reports of complete AIV genomes with a Eurasian lineage in North America or complete AIV genomes with an American lineage in Eurasia or Africa [12], [25]. Based on analysis of AIV sequences deposited in public repositories, movements of AIVs between the Americas and Eurasia is rare [12], [25], consistent with the known inverse effect of geographic distance and AIV gene flow [26]. Even though the frequency of AIV detection is much higher in waterfowl, cases of one or more segments of European lineage viruses being detected in the Americas or one or more segments of American lineage viruses detected in Eurasia are more common in gulls and shorebirds [19], [25].

In this study we examined the North Atlantic as a potential route for migratory birds to transport AIVs between North America and Europe. Iceland is located along the East Atlantic Flyway and a number of species of migratory birds stopover there during annual spring and autumn migrations between North America and Europe and Africa. Previous to this study there were published reports of only 2 AIVs from wild birds in Iceland, and only a single segment sequence from one of those viruses is available in public repositories (A/teal/Iceland/29/80) [27], [28]. Herein we report the genomic sequences of 29 AIVs obtained from wild birds in Iceland and demonstrate the importance of the North Atlantic in the intercontinental movement of AIV and the intermixing of the major AIV lineages.

## Results

**Figure 1:**
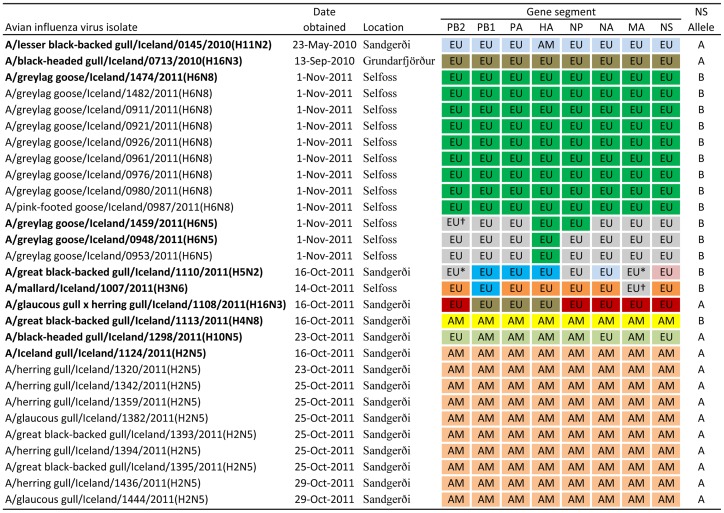
Viruses recovered from Iceland wild birds in 2010–2011 with segment lineage detail. EU denotes the segment is most similar to Eurasian lineage viruses, AM denotes the segment is most similar to American lineage viruses. Within each segment column, segments that have the same color indicate a ≥99% nucleotide sequence similarity among that segment. * indicates a ≥99% similarity to only one other virus segment of the same color denoted by †. Bold text indicates novel virus assemblages.

We sampled 1078 waterfowl, gulls, and shorebirds in Iceland (Table S1) and isolated 29 AIVs (Prevalence  = 2.9%). Analysis of genome sequences identified 8 HA and 5 NA subtypes and a total of 11 unique viruses after grouping highly similar (≥99%) viral genotypes (Figure 1). In combination, we isolated 10 H2N5, 9 H6N8, 3 H6N5, 2 H16N3, and single isolates of H3N6, H4N8, H5N2, H10N5, and H11N2 subtypes. The H6N5 and H16N3 viruses had 2 different genotypes each. The nucleotide sequences from the 10 H2N5 and 9 H6N8 viruses were highly similar within each subtype and are considered single occurrences of both subtypes.

The subtypes H6N5 and H6N8 were detected in graylag geese (*Anser anser*) along with a single detection of H6N8 in a pink-footed goose (*Anser brachyrhynchus*; Table S1). The single H3N6 subtype was detected in a mallard. The remaining virus subtypes were detected in various gull species, including black-headed gull (*Chroicocephalus ridibundus*), lesser black-backed gull (*Larus fuscus*), great black-backed gull (*Larus marinus*), glaucous gull (*Larus hyperboreus*), herring gull (*Larus argentatus*), Iceland gull (*Larus glaucoides*), and a single hybrid glaucous x herring gull (*Larus hyperboreus x Larus argentatus*, Table S1).

Phylogenetic and BLAST analyses of the 8 segments that made up each of 11 unique viruses revealed 2 viruses of completely American lineage, 7 that were completely Eurasian lineage, and 2 that were mixed American and Eurasian lineage (Figure 1, Figures S1, S2, S3, S4, S5, S6, S7, S8, S9, S10, S11, S12, S13, S14, S15, S16, S17, S18, S19). Phylogenetic trees were inferred from Maximum Likelihood analysis.

### American lineage viruses

After grouping highly related viruses we detected 2 distinct AIVs that were entirely American lineage viruses and included all the H2N5 and H4N8 virus subtypes isolated. In these 2 viruses, all 8 segments were most closely related to American lineage viruses and fell within previously identified American clades [14], [15]. Both viruses (along with 2 Eurasian lineage viruses) were first detected in a mixed flock of gulls captured on the same day. The H4N8 subtype and the 2 Eurasian lineage viruses were not detected again in our sampling effort, however, the H2N5 subtype was detected in gulls at subsequent captures over a period of 14 days (Oct 16, 23, 25, and 29). The 2 American lineage viruses did not have any highly similar segments in common (Figure 1).

### Eurasian lineage viruses

We isolated 7 distinct AIVs that were entirely of Eurasian lineage after grouping highly related viruses. All of the segments from AIVs obtained from geese and the single virus from a mallard were of Eurasian lineage. In addition, 5 gull species had AIVs of completely Eurasian lineage. In these 7 viruses, all 8 segments were most closely related to Eurasian lineage viruses and fell within previously identified Eurasian clades [14], [15]. Of the Eurasian lineage viruses, several had highly similar segments (Figure 1). For example, of the 2 H16N3 virus subtypes isolated from gulls, 3 of 8 segments were highly similar (≥99%).

We isolated a single H5 virus subtype from a great black-backed gull and determined that the proteolytic cleavage site of the HA segment contained a cleavage site motif (RETR/GLF), consistent with LPAIV [29], [30].

### Mixed American and Eurasian lineage virus

We detected 2 viruses, an H10N5 from a black-headed gull and an H11N2 from a lesser black-backed gull, that contained mixed lineage genomes. The H10N5 virus contained 5 segments (PB1, PA, HA, NP, and M) that were genetically most closely related to previously identified American lineage viruses, with the remaining 3 segments (PB2, NA, and NS) more closely related to previously identified Eurasian lineage segments [14], [15]. The H11N2 subtype contained 7 segments (PB2, PB1, PA, NP, NA, M, and NS) that were genetically most closely related to previously identified Eurasian lineage viruses but the HA segment was most closely related to American lineage viruses [14], [15]. The NA segment of this virus was highly similar to the NA segment of the Icelandic H5N2 virus we isolated from a great black-backed gull (Figure 1).

### NS allele diversity

Of the 11 unique virus genomes recovered from Iceland, 5 contained NS segments that belonged to allele A lineage and 6 contained NS segments that belonged to allele B lineage. Each NS allele was also represented in both Eurasian and American lineage viruses (Figure S6). The allele A lineage viruses included the 2 H16N3 virus subtypes that were of Eurasian gull lineage, the H10N5 and H11N2 virus subtypes were of Eurasian waterfowl lineage, and the H2N5 virus subtype were of an American waterfowl lineage (Figure 1). Of the 6 allele B viruses, the H4N8 subtype was of American lineage and the remaining virus subtypes (H3N6, H5N2, 2 H6N5, and H6N8) were all of Eurasian lineage (Figure 1).

## Discussion

**Figure 2:**
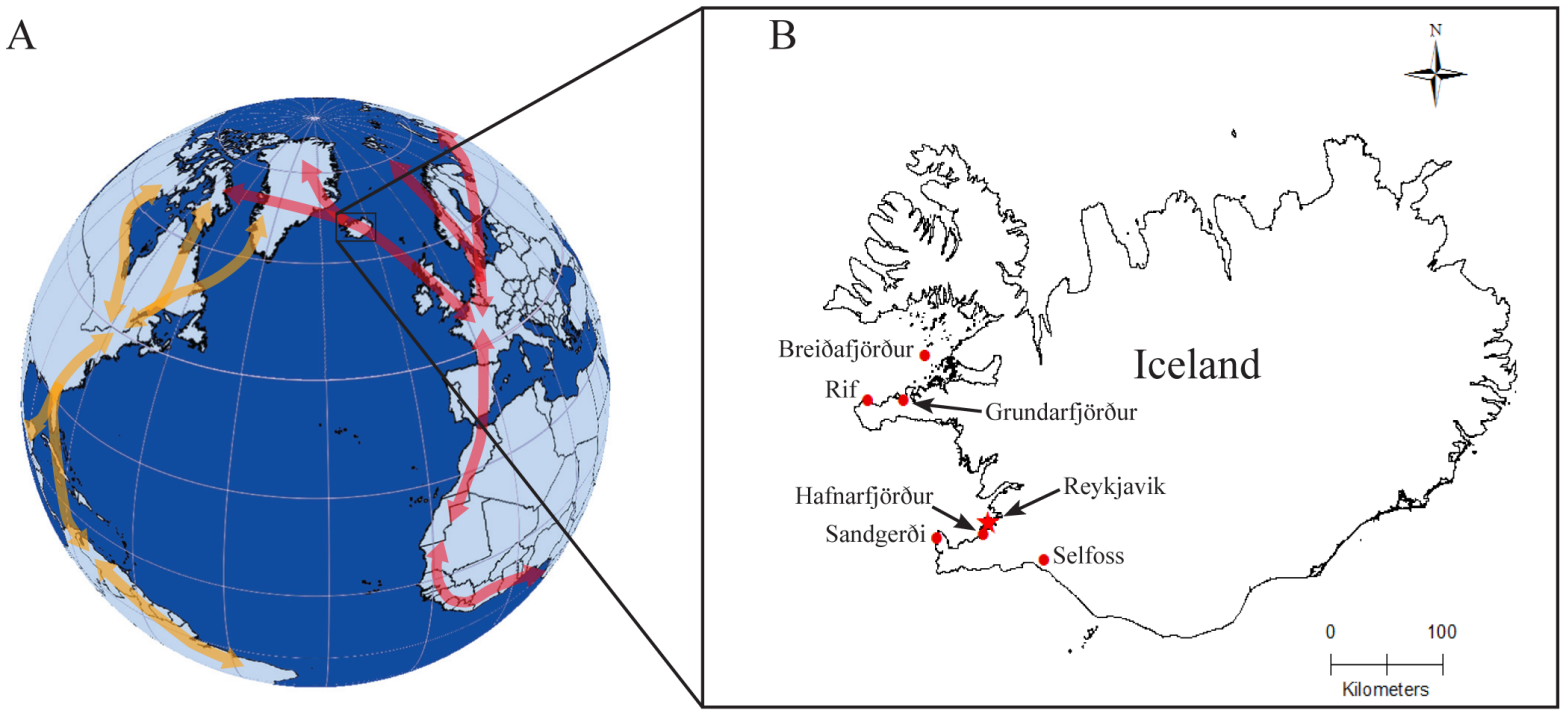
A. Map showing position of Iceland relative to the East Atlantic Flyway (red arrows) and the North American Atlantic Flyway (yellow arrows). Flyways represent generalized migration movements of birds with most using only portions of the flyways. Actual regions of flyways used by migratory birds are dependent on species and breeding population. B. Map of Iceland depicting bird sampling locations (red dots) used in this study and Reykjavik (red star) is provided for reference. Breiðafjörður and Selfoss sampling locations are generalized as samples provided by hunters and fisherman were obtained over a larger area within these marked regions.

In this study we isolated 11 unique AIVs in gulls and waterfowl from Iceland that contained unexpectedly high viral genetic diversity. Most significantly, we obtained viruses that were completely (all 8 segments) of Eurasian lineage or American lineage as well as reassortant American and Eurasian lineage viruses. Previous to this study there have been no reports of complete American or Eurasian lineage viruses in the same geographic location [12], [25]. When Eurasian AIV segments have been detected in the Americas, or American AIV segments in Eurasia, it has generally been only 1 or 2 segments per virus [12], [18], [19], [25]. However, 2 recent studies in North America have found viruses with near complete (7 of 8 segments) Eurasian lineage genomes [16], [19]. Both of these studies were conducted in regions where predominately Eurasian flyways overlap into North America (the East Asian Flyway and the East Atlantic Flyway). Iceland is within the East Atlantic Flyway (Figure 2); this flyway extends into North America (Greenland and eastern Canada) and tens of thousands of migratory birds move from North America into Europe along this route on their way to and from breeding and non-breeding grounds [31], [32]. Our data demonstrate that the North Atlantic serves as a route for intercontinental movement of AIV and it will be important to track the further dissemination of these viruses, in whole, or in part, into the Icelandic avian community and, more significantly, into the avian communities of Europe or North America.

During our analysis we noted that our H4N8 virus obtained from a great black-backed gull in Iceland was nearly identical to an H4N8 virus obtained from a bufflehead (*Bucephala albeola*) sampled in Maine, U.S.A. These two isolates were obtained approximately 1 month apart and field samples were tested in the same laboratory with both isolates being sent to the same sequencing laboratory. It is unusual to find two influenza viruses that are nearly identical in all the nucleotides of the consensus sequence. To determine if this was a potential contaminant we reviewed all laboratory procedures and performed single nucleotide position (SNP) analysis on the sequencing results. SNP analysis did identify a single minor SNP variation in the A/great black-backed gull/Iceland/1113/2011(H4N8) at position 174 of the NA (541 reads show A, 84 reads show G) that is not present in the other virus, which suggests the viral genomic information was derived from different specimens.

We found the highest diversity of viruses in gulls, and all the AIV lineage diversity we detected in Iceland was represented within this avian group. In contrast, viruses recovered from waterfowl in Iceland contained only segments that were of Eurasian waterfowl lineage and NS allele B. Previous studies have made similar observations. In Alaska, glaucous-winged gulls (*Larus glaucescens*) were the host species in which an AIV was obtained that contained 7 of 8 segments of Eurasian lineage [19]. In eastern Canada, the host species that contained a virus with 7 segments of Eurasian lineage was a mallard but the segments were most closely related to Eurasian gull lineages [16]. In addition, analysis of AIVs in public sequence databases for outsider events (where single or multiple segments from an AIV found in Eurasia are of American lineage, or vice versa) found these events predominantly detected from viruses obtained from gulls and shorebirds [12], [19], [25]. Our data reinforce the idea that gulls are more important in the intercontinental movement of AIV than previously thought [16], [19], [20].

The phylogenetic analysis we conducted supported previous reports of gull specific lineages particularly for the NP, M, and NS genes [19]. Our analysis also detected a gull lineage that is independent of the American and European lineages in the NA N3 subtype. This gull lineage consists of viruses obtained from gulls in North America, Europe, and Asia and is distinct from other viruses obtained from gulls and waterfowl (Figure S16). The first gull we detected the American lineage H2N5 virus in was an Iceland gull, a species that breeds in Greenland and northeastern Canada and predominantly winters in the North Atlantic, with an estimated 5,000 to 10,000 individuals wintering along the coast of Iceland [33]. Great black-backed gull, another species we found with a completely American lineage AIV, breeds throughout the North Atlantic but has been shown to make transatlantic movements [20], [33]. While we cannot be certain that the American lineage viruses we detected were brought to Iceland by gulls, at least one of these American viruses was circulating within the gull population during our sampling period as it was found in 10 different birds over a period of 14 days. The ranges of gull species in the North Atlantic, especially those in Iceland, are well known, but the connectivity of these populations is poorly understood except in a few cases [33], [34]. However, it is possible that there is greater connectivity between populations of other species of gulls than has been appreciated [20], [35].

Iceland is in a unique geographic situation possibly making detection of highly diverse and reassortant AIVs more likely. With few other stopover sites available in this region, Iceland provides refuge for migrants during unfavorable weather conditions as well as important feeding areas for birds to rebuild critical energy reserves to continue migration [36]. In addition, birds infected with AIV just prior to migration may be prone to shorter migratory distances and longer stopovers during migration, increasing the chance that AIV infected birds will stopover in Iceland [2].

Our data demonstrate that Iceland represents an important location for the transmission of AIV among wild birds and that gulls play a much larger role in the intercontinental movement of AIV than they have been previously represented in surveillance programs. Intriguingly, we found more AIV diversity in gulls than in waterfowl. More importantly, gull and waterfowl lineage AIVs, with entirely American and Eurasian lineage AIVs as well as mixed lineage AIVs, were identified in gulls. Gulls, as a taxonomic group, are globally distributed; however, much of what is known about gulls in relation to AIV is limited to the northern hemisphere. Gulls at mid-latitudes and in the southern hemisphere have similar migration strategies and movements to northern hemisphere gulls but have only infrequently been found infected with AIV [37]–[40]. Based on our observations there is a need to continue to examine migration patterns of gulls and the connectivity of gull populations globally. Documentation of reassortment and generation of new AIV genetic combinations in Icelandic avian hosts as well as intercontinental virus movements also highlights the need for continued surveillance of AIV and other pathogens in Iceland and elsewhere in the North Atlantic.

## Methods

### Ethics statement

This research was conducted under approval of the U.S. Geological Survey National Wildlife Health Center's Animal Care and Use Committee, protocol number #EP090325, in strict accordance with guidelines set forth in the U.S. Governments Animal Welfare Act and the National Institutes of Health Office of Laboratory Animal Welfare. Permits for the capture and sampling of wild birds were issued by the Icelandic Institute of Natural History (Permit Numbers 368 and 403). Permits to ship collected cloacal swab samples from Iceland to the United States (US) were obtained from the Icelandic Institute of Natural History, the US Department of Agricultural, and the US Fish and Wildlife Service. No CITES (Convention on International Trade in Endangered Species) protected species were sampled. Except at two sites where birds were captured on private property, all sampling occurred in conjunction with the Southwest Iceland Nature Research Institute and/or the University of Iceland, Snæfellsnes Research Centre on public land within regulations of these institutes and the Icelandic Institute of Natural History. Capture of birds on private property was carried out with the land owners' specific permission.

### Field sampling

Between May 17–30, 2010, September 5–13, 2010, and October 14- November 1, 2011 gulls and shorebirds were live-captured using a 18 m×12 m cannon-propelled capture net. Gulls were captured at baited locations in Sandgerði (Lat. 64.039°, Long. −22.712°; May 2010, Oct 2011), Hafnarfjörður (Lat. 64.063°, Long. −21.981°; September 2010), and Grundarfjörður (Lat. 64.924°, Long. −23.254°; September 2010), Iceland (Figure 2). Shorebirds were captured at roost sites on beaches from Sandgerði north to Garðskagi (May, 2010), and Hafnarfjörður (September 2010), Iceland. In addition, shorebirds already attending nests in May, 2010 at Sandgerði, Iceland were live-captured using walk-in traps placed over the nest. In June 2010 we used noose poles to live-capture colony nesting common eiders (*Somateria mollissima*) in Rif, Iceland (Lat. 64.920°, Long. −23.822°; Figure 2). All live-captured birds were marked with metal bands, had cloacal swab samples collected, and then were immediately released. In June and July 2010 common eiders and various seabirds were obtained from fisherman at Breiðafjörður (Lat. 65.2°, Long. −22.9°) that were dead as a result of commercial fishing activities (Figure 2). We also sampled hunter-killed geese and ducks near Selfoss, Iceland (Lat. 63.9°, Long. −21.0°, Oct 2011; Figure 2).

Each cloacal swab sample was immediately placed in individual 2.0 ml cryovials containing 1.25 ml viral transport media [41]. Vials were held on ice for up to 5 hours prior to being stored in liquid nitrogen or liquid nitrogen vapor. Shipment of samples in biosecure containers from Iceland to Madison, Wisconsin, USA was by private courier with samples stored on dry ice. Once received in the laboratory, samples were stored at −80C until analysis.

### Laboratory analysis and sequencing

Viral RNA was extracted using the MagMAX™-96 AI/ND Viral RNA Isolation Kit (Ambion, Austin, TX) following the manufacturer's procedures. Real time RT-PCR was performed using previously published procedures, primers, and probe designed to detect the influenza A virus M gene [42]. Virus isolation was performed in embryonated chicken eggs on all samples collected in 2010 and 2011 exhibiting positive Ct values from RT-PCR analysis [43]. In 2011 virus isolation was additionally performed on all RT-PCR negative samples.

Virus isolates were submitted to the J. Craig Venter Institute under contract with the NIH/NIAID where the viral RNA was isolated and the genomes amplified using a universal multisegment RT-PCR procedure and sequenced using a high throughput next-generation sequencing (Illumina HiSeq 2000 and Roche 454) assembly pipeline and established protocols [44]–[46]. Virus segments that had ≥99% nucleotide sequence similarity with the same segments from other viruses obtained from this study were considered highly similar. Viruses in which all segments had ≥99% nucleotide sequence identity with the same segments from viruses obtained from this study were considered the same virus. Sequences were deposited in GenBank with accession numbers CY138137-CY138152 and CY149332-CY149547.

For phylogenetic analysis, sequences were aligned using ClustalW with representative Eurasian and North American viruses, the most similar virus in GenBank as determined by BLAST analysis (Table S2), and selected contemporary sequences that were closely related to the Icelandic viruses we isolated as determined by GenBank [14], [15], [47], [48]. Phylogenetic and molecular evolutionary analyses were conducted using *MEGA* version 5.05 [49]. The evolutionary history was inferred using the Maximum Likelihood method using nearest neighbor interchange with the Tamura-Nei substitution model assuming uniform rates and 1000 bootstrap replicates [50], [51]. All positions containing gaps and missing data were deleted.

## Supporting Information

Figure S1 **Maximum Likelihood analysis of avian influenza virus segment PB2.** Evolutionary history was inferred using the Maximum Likelihood analysis using the Tamura-Nei substitution model in Mega 5.05. A total of 1000 bootstrap replicates were used. Percentages of replicate trees (when ≥50%) in which the associated taxa clustered together are shown next to the branches. The tree is drawn to scale, with branch lengths measured in the number of substitutions per site. Red diamonds indicate Iceland isolates.
(PDF)

Figure S2 **Maximum Likelihood analysis of avian influenza virus segment PB1.** Evolutionary history was inferred using the Maximum Likelihood analysis using the Tamura-Nei substitution model in Mega 5.05. A total of 1000 bootstrap replicates were used. Percentages of replicate trees (when ≥50%) in which the associated taxa clustered together are shown next to the branches. The tree is drawn to scale, with branch lengths measured in the number of substitutions per site. Red diamonds indicate Iceland isolates.
(PDF)

Figure S3 **Maximum Likelihood analysis of avian influenza virus segment PA.** Evolutionary history was inferred using the Maximum Likelihood analysis using the Tamura-Nei substitution model in Mega 5.05. A total of 1000 bootstrap replicates were used. Percentages of replicate trees (when ≥50%) in which the associated taxa clustered together are shown next to the branches. The tree is drawn to scale, with branch lengths measured in the number of substitutions per site. Red diamonds indicate Iceland isolates.* American lineage 3 and ** American lineage as described by Bahl et al. (2009).
(PDF)

Figure S4 **Maximum Likelihood analysis of avian influenza virus segment NP.** Evolutionary history was inferred using the Maximum Likelihood analysis using the Tamura-Nei substitution model in Mega 5.05. A total of 1000 bootstrap replicates were used. Percentages of replicate trees (when ≥50%) in which the associated taxa clustered together are shown next to the branches. The tree is drawn to scale, with branch lengths measured in the number of substitutions per site. Red diamonds indicate Iceland isolates.
(PDF)

Figure S5 **Maximum Likelihood analysis of avian influenza virus segment M.** Evolutionary history was inferred using the Maximum Likelihood analysis using the Tamura-Nei substitution model in Mega 5.05. A total of 1000 bootstrap replicates were used. Percentages of replicate trees (when ≥50%) in which the associated taxa clustered together are shown next to the branches. The tree is drawn to scale, with branch lengths measured in the number of substitutions per site. Red diamonds indicate Iceland isolates.
(PDF)

Figure S6 **Maximum Likelihood analysis of avian influenza virus segment NS.** Evolutionary history was inferred using the Maximum Likelihood analysis using the Tamura-Nei substitution model in Mega 5.05. A total of 1000 bootstrap replicates were used. Percentages of replicate trees (when ≥50%) in which the associated taxa clustered together are shown next to the branches. The tree is drawn to scale, with branch lengths measured in the number of substitutions per site. Red diamonds indicate Iceland isolates.
(PDF)

Figure S7 **Maximum Likelihood analysis of avian influenza virus segment HA H2.** Evolutionary history was inferred using the Maximum Likelihood analysis using the Tamura-Nei substitution model in Mega 5.05. A total of 1000 bootstrap replicates were used. Percentages of replicate trees (when ≥50%) in which the associated taxa clustered together are shown next to the branches. The tree is drawn to scale, with branch lengths measured in the number of substitutions per site. Red diamonds indicate Iceland isolates.
(PDF)

Figure S8 **Maximum Likelihood analysis of avian influenza virus segment HA H3.** Evolutionary history was inferred using the Maximum Likelihood analysis using the Tamura-Nei substitution model in Mega 5.05. A total of 1000 bootstrap replicates were used. Percentages of replicate trees (when ≥50%) in which the associated taxa clustered together are shown next to the branches. The tree is drawn to scale, with branch lengths measured in the number of substitutions per site. Red diamonds indicate Iceland isolates.
(PDF)

Figure S9 **Maximum Likelihood analysis of avian influenza virus segment HA H4.** Evolutionary history was inferred using the Maximum Likelihood analysis using the Tamura-Nei substitution model in Mega 5.05. A total of 1000 bootstrap replicates were used. Percentages of replicate trees (when ≥50%) in which the associated taxa clustered together are shown next to the branches. The tree is drawn to scale, with branch lengths measured in the number of substitutions per site. Red diamonds indicate Iceland isolates.
(PDF)

Figure S10 **Maximum Likelihood analysis of avian influenza virus segment HA H5.** Evolutionary history was inferred using the Maximum Likelihood analysis using the Tamura-Nei substitution model in Mega 5.05. A total of 1000 bootstrap replicates were used. Percentages of replicate trees (when ≥50%) in which the associated taxa clustered together are shown next to the branches. The tree is drawn to scale, with branch lengths measured in the number of substitutions per site. Red diamonds indicate Iceland isolates.
(PDF)

Figure S11 **Maximum Likelihood analysis of avian influenza virus segment HA H6.** Evolutionary history was inferred using the Maximum Likelihood analysis using the Tamura-Nei substitution model in Mega 5.05. A total of 1000 bootstrap replicates were used. Percentages of replicate trees (when ≥50%) in which the associated taxa clustered together are shown next to the branches. The tree is drawn to scale, with branch lengths measured in the number of substitutions per site. Red diamonds indicate Iceland isolates.
(PDF)

Figure S12 **Maximum Likelihood analysis of avian influenza virus segment HA H10.** Evolutionary history was inferred using the Maximum Likelihood analysis using the Tamura-Nei substitution model in Mega 5.05. A total of 1000 bootstrap replicates were used. Percentages of replicate trees (when ≥50%) in which the associated taxa clustered together are shown next to the branches. The tree is drawn to scale, with branch lengths measured in the number of substitutions per site. Red diamonds indicate Iceland isolates.
(PDF)

Figure S13 **Maximum Likelihood analysis of avian influenza virus segment HA H11.** Evolutionary history was inferred using the Maximum Likelihood analysis using the Tamura-Nei substitution model in Mega 5.05. A total of 1000 bootstrap replicates were used. Percentages of replicate trees (when ≥50%) in which the associated taxa clustered together are shown next to the branches. The tree is drawn to scale, with branch lengths measured in the number of substitutions per site. Red diamonds indicate Iceland isolates.
(PDF)

Figure S14 **Maximum Likelihood analysis of avian influenza virus segment HA H16.** Evolutionary history was inferred using the Maximum Likelihood analysis using the Tamura-Nei substitution model in Mega 5.05. A total of 1000 bootstrap replicates were used. Percentages of replicate trees (when ≥50%) in which the associated taxa clustered together are shown next to the branches. The tree is drawn to scale, with branch lengths measured in the number of substitutions per site. Red diamonds indicate Iceland isolates.
(PDF)

Figure S15 **Maximum Likelihood analysis of avian influenza virus segment NA N2.** Evolutionary history was inferred using the Maximum Likelihood analysis using the Tamura-Nei substitution model in Mega 5.05. A total of 1000 bootstrap replicates were used. Percentages of replicate trees (when ≥50%) in which the associated taxa clustered together are shown next to the branches. The tree is drawn to scale, with branch lengths measured in the number of substitutions per site. Red diamonds indicate Iceland isolates.
(PDF)

Figure S16 **Maximum Likelihood analysis of avian influenza virus segment NA N3.** Evolutionary history was inferred using the Maximum Likelihood analysis using the Tamura-Nei substitution model in Mega 5.05. A total of 1000 bootstrap replicates were used. Percentages of replicate trees (when ≥50%) in which the associated taxa clustered together are shown next to the branches. The tree is drawn to scale, with branch lengths measured in the number of substitutions per site. Red diamonds indicate Iceland isolates.
(PDF)

Figure S17 **Maximum Likelihood analysis of avian influenza virus segment NA N5.** Evolutionary history was inferred using the Maximum Likelihood analysis using the Tamura-Nei substitution model in Mega 5.05. A total of 1000 bootstrap replicates were used. Percentages of replicate trees (when ≥50%) in which the associated taxa clustered together are shown next to the branches. The tree is drawn to scale, with branch lengths measured in the number of substitutions per site. Red diamonds indicate Iceland isolates.
(PDF)

Figure S18 **Maximum Likelihood analysis of avian influenza virus segment NA N6.** Evolutionary history was inferred using the Maximum Likelihood analysis using the Tamura-Nei substitution model in Mega 5.05. A total of 1000 bootstrap replicates were used. Percentages of replicate trees (when ≥50%) in which the associated taxa clustered together are shown next to the branches. The tree is drawn to scale, with branch lengths measured in the number of substitutions per site. Red diamonds indicate Iceland isolates.
(PDF)

Figure S19 **Maximum Likelihood analysis of avian influenza virus segment NA N8.** Evolutionary history was inferred using the Maximum Likelihood analysis using the Tamura-Nei substitution model in Mega 5.05. A total of 1000 bootstrap replicates were used. Percentages of replicate trees (when ≥50%) in which the associated taxa clustered together are shown next to the branches. The tree is drawn to scale, with branch lengths measured in the number of substitutions per site. Red diamonds indicate Iceland isolates.
(PDF)

Table S1 **Total cloacal swabs obtained and total virus positive for avian influenza virus from wild birds in Iceland, 2010–2011.** Apparent prevalence for those species with viruses isolated shown in parenthesis.
(DOCX)

Table S2 **Results of standard nucleotide Basic Local Allignment Search Tool (BLAST) conducted using GenBank for each virus segment we detected in Iceland, 2010–2011.**
(XLSX)
