# Supplementary figures and images for: North Atlantic Migratory Bird Flyways Provide Routes for Intercontinental Movement of Avian Influenza Viruses
Source: PLoS One. 2014 Mar 19;9(3):e92075. doi: 10.1371/journal.pone.0092075 (PMC3960164; doi:10.1371/journal.pone.0092075)

---

0.02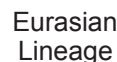

## American Lineage

Supplement: Figure S1 — Maximum Likelihood analysis of avian influenza virus segment PB2. Evolutionary history was inferred using the Maximum Likelihood analysis using the Tamura-Nei substitution model in Mega 5.05. A total of 1000 bootstrap replicates were used. Percentages of replicate trees (when ≥50%) in which the associated taxa clustered together are shown next to the branches. The tree is drawn to scale, with branch lengths measured in the number of substitutions per site. Red diamonds indicate Iceland isolates. (PDF) [file pone.0092075.s001.pdf]

PB1

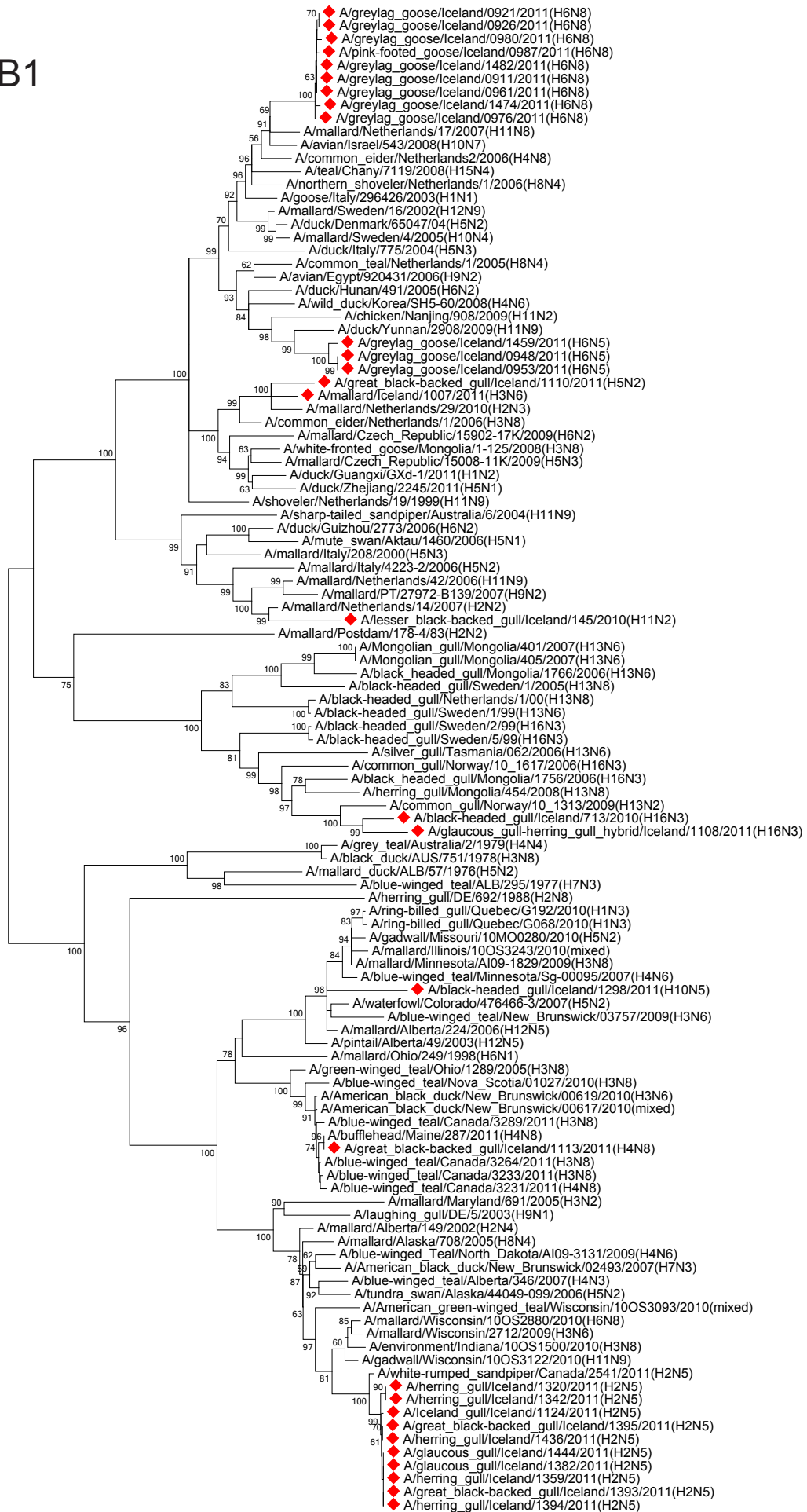

0.01

Supplement: Figure S2 — Maximum Likelihood analysis of avian influenza virus segment PB1. Evolutionary history was inferred using the Maximum Likelihood analysis using the Tamura-Nei substitution model in Mega 5.05. A total of 1000 bootstrap replicates were used. Percentages of replicate trees (when ≥50%) in which the associated taxa clustered together are shown next to the branches. The tree is drawn to scale, with branch lengths measured in the number of substitutions per site. Red diamonds indicate Iceland isolates. (PDF) [file pone.0092075.s002.pdf]

PA

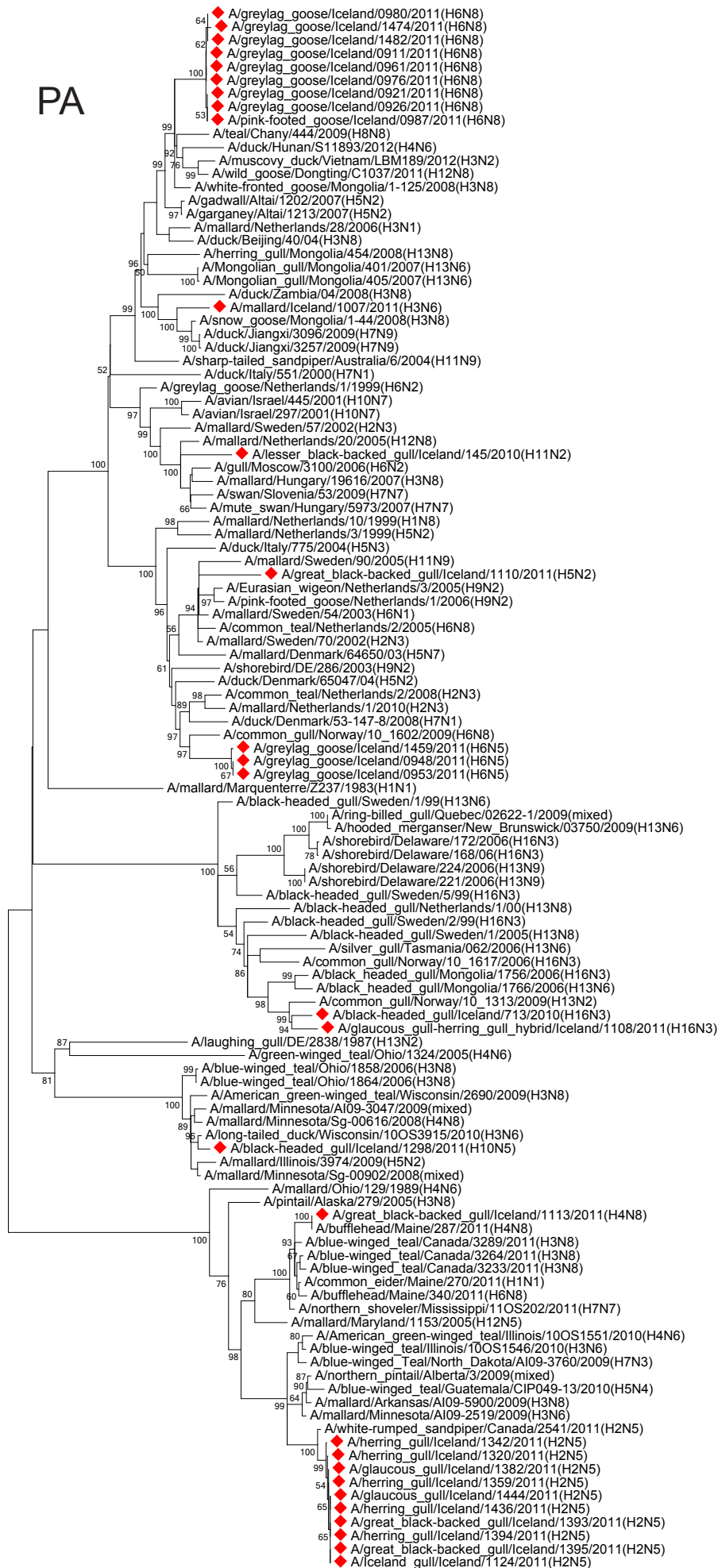

0.02

Supplement: Figure S3 — Maximum Likelihood analysis of avian influenza virus segment PA. Evolutionary history was inferred using the Maximum Likelihood analysis using the Tamura-Nei substitution model in Mega 5.05. A total of 1000 bootstrap replicates were used. Percentages of replicate trees (when ≥50%) in which the associated taxa clustered together are shown next to the branches. The tree is drawn to scale, with branch lengths measured in the number of substitutions per site. Red diamonds indicate Iceland isolates.* American lineage 3 and ** American lineage as described by Bahl et al. (2009). (PDF) [file pone.0092075.s003.pdf]

NP

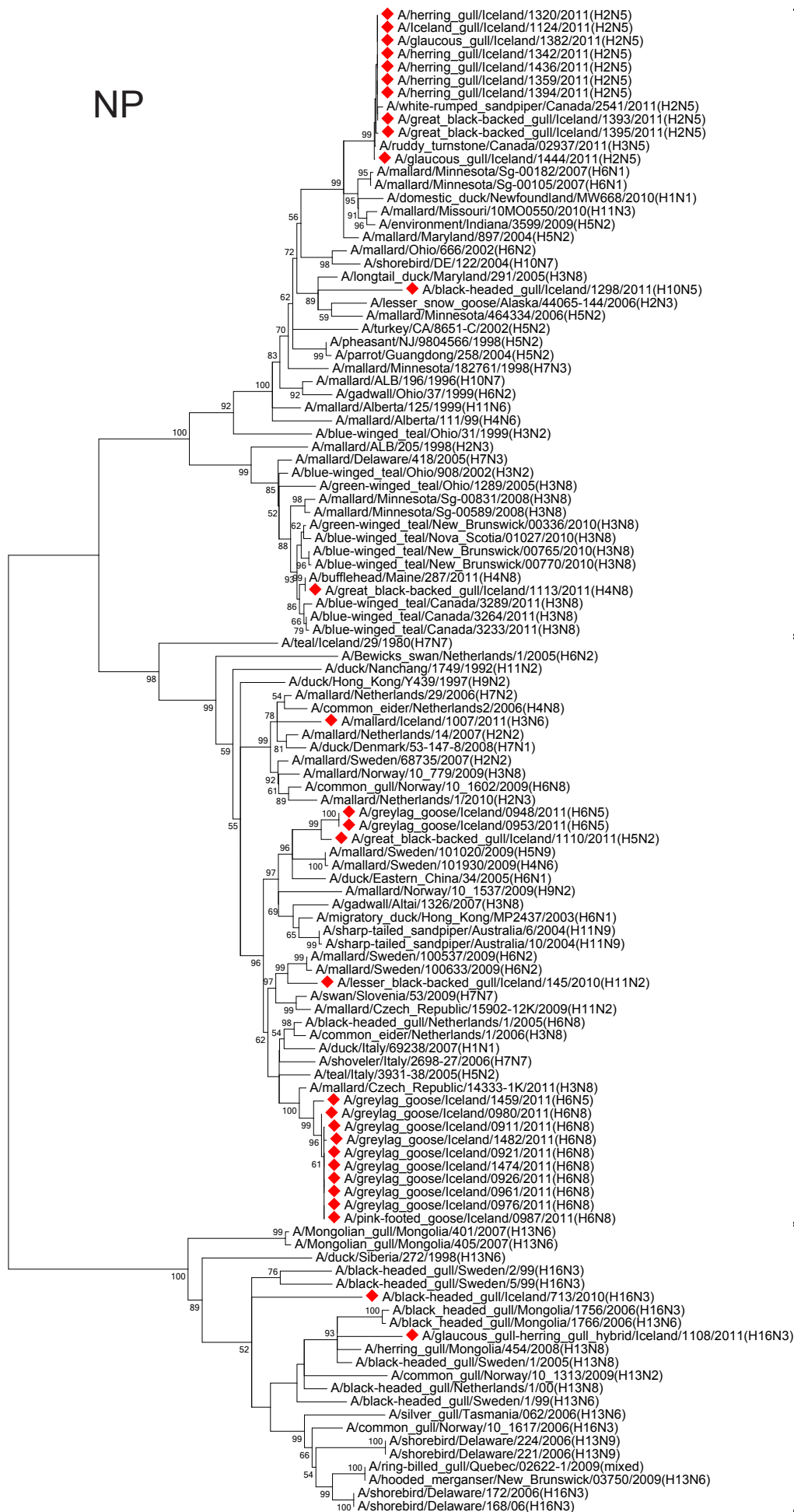

0.02

Supplement: Figure S4 — Maximum Likelihood analysis of avian influenza virus segment NP. Evolutionary history was inferred using the Maximum Likelihood analysis using the Tamura-Nei substitution model in Mega 5.05. A total of 1000 bootstrap replicates were used. Percentages of replicate trees (when ≥50%) in which the associated taxa clustered together are shown next to the branches. The tree is drawn to scale, with branch lengths measured in the number of substitutions per site. Red diamonds indicate Iceland isolates. (PDF) [file pone.0092075.s004.pdf]

0.01

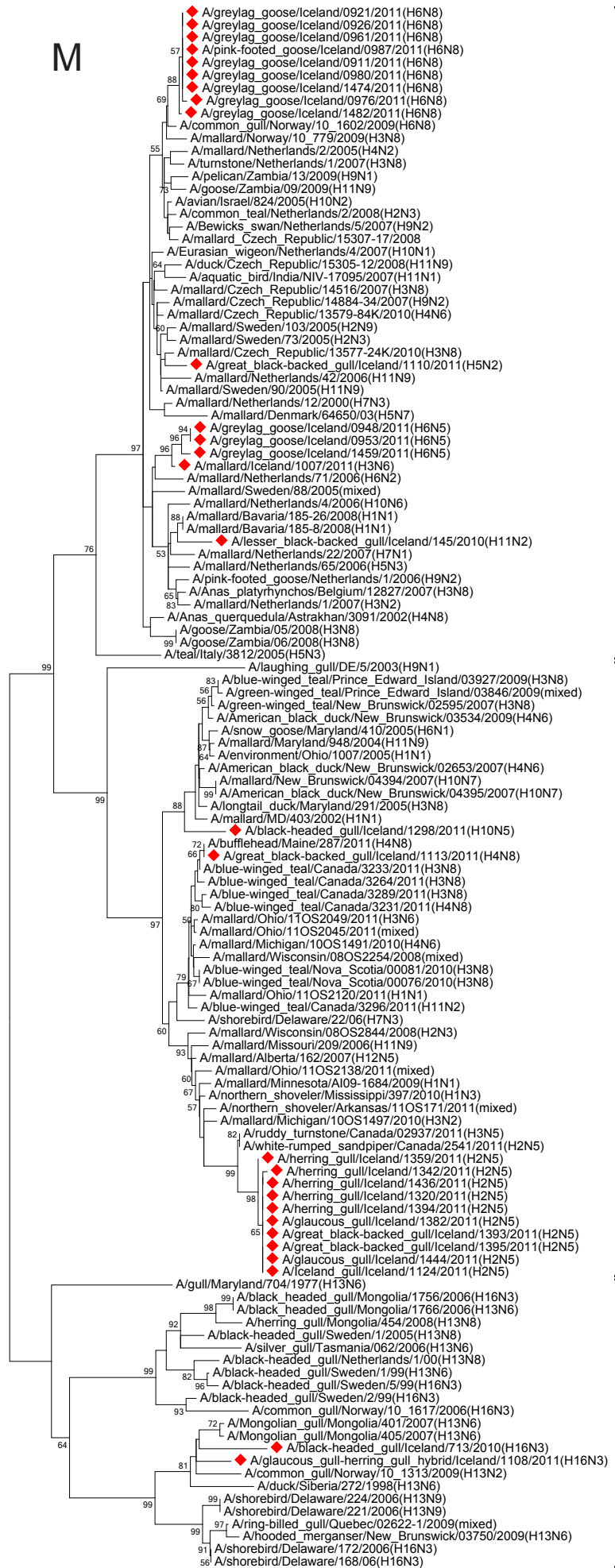

Supplement: Figure S5 — Maximum Likelihood analysis of avian influenza virus segment M. Evolutionary history was inferred using the Maximum Likelihood analysis using the Tamura-Nei substitution model in Mega 5.05. A total of 1000 bootstrap replicates were used. Percentages of replicate trees (when ≥50%) in which the associated taxa clustered together are shown next to the branches. The tree is drawn to scale, with branch lengths measured in the number of substitutions per site. Red diamonds indicate Iceland isolates. (PDF) [file pone.0092075.s005.pdf]

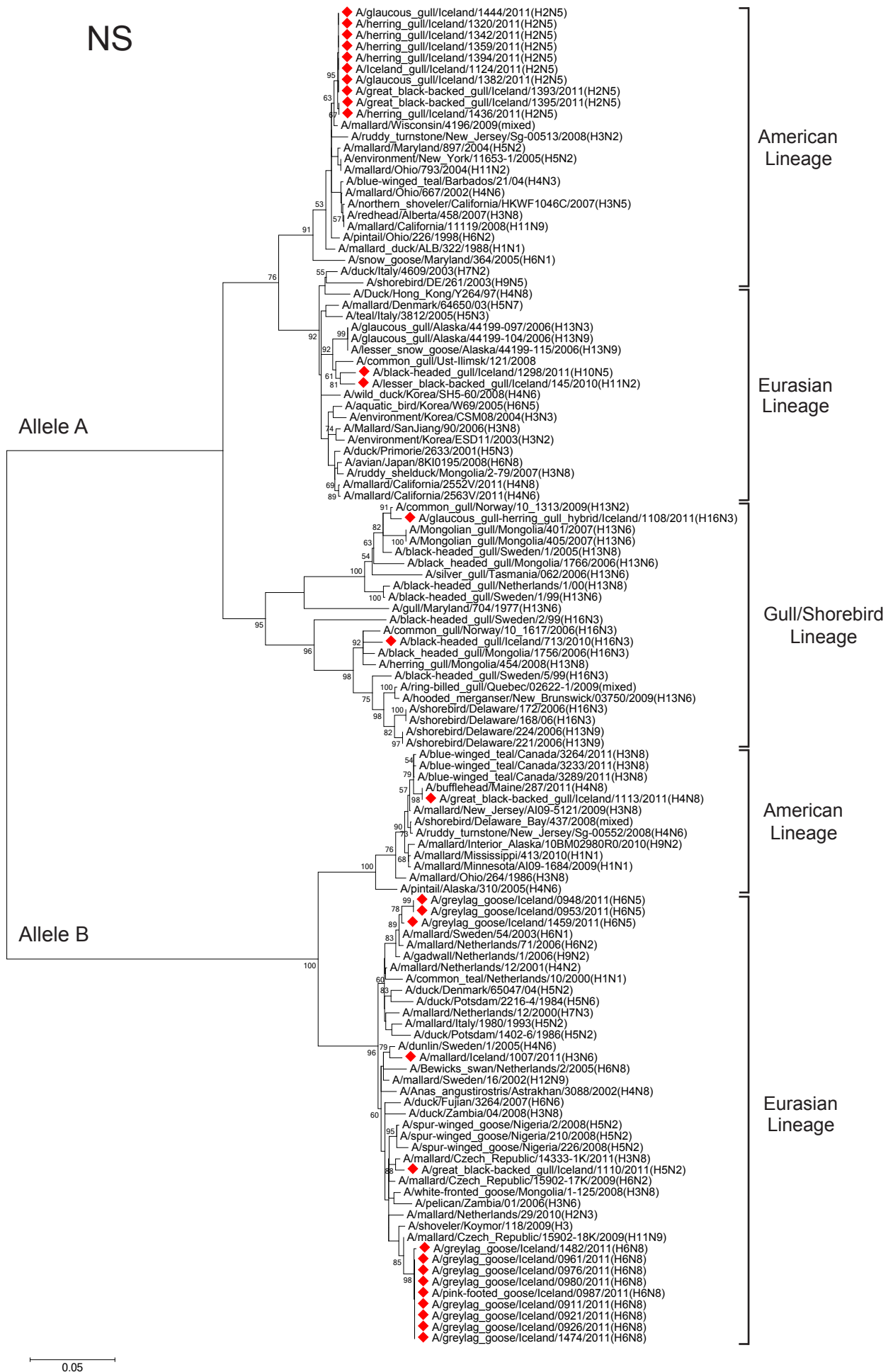

Supplement: Figure S6 — Maximum Likelihood analysis of avian influenza virus segment NS. Evolutionary history was inferred using the Maximum Likelihood analysis using the Tamura-Nei substitution model in Mega 5.05. A total of 1000 bootstrap replicates were used. Percentages of replicate trees (when ≥50%) in which the associated taxa clustered together are shown next to the branches. The tree is drawn to scale, with branch lengths measured in the number of substitutions per site. Red diamonds indicate Iceland isolates. (PDF) [file pone.0092075.s006.pdf]

## HA H2

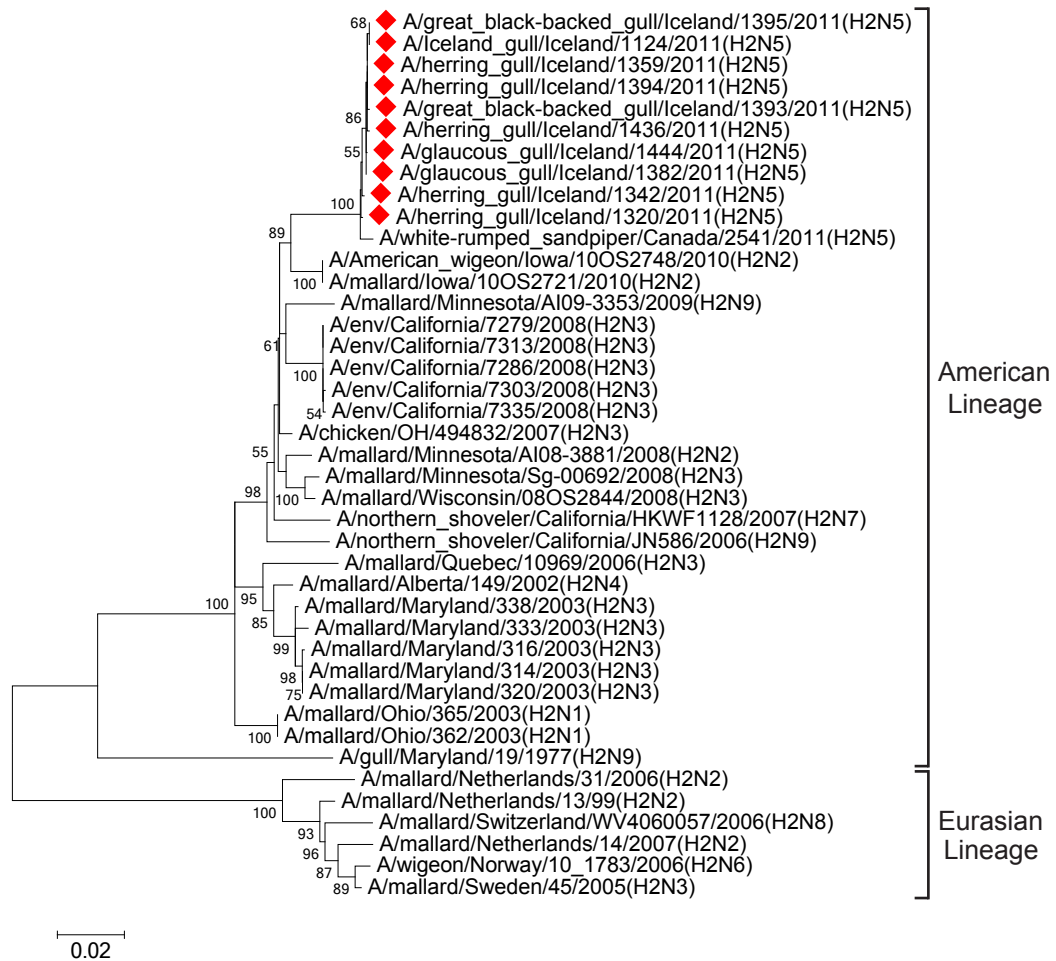

Supplement: Figure S7 — Maximum Likelihood analysis of avian influenza virus segment HA H2. Evolutionary history was inferred using the Maximum Likelihood analysis using the Tamura-Nei substitution model in Mega 5.05. A total of 1000 bootstrap replicates were used. Percentages of replicate trees (when ≥50%) in which the associated taxa clustered together are shown next to the branches. The tree is drawn to scale, with branch lengths measured in the number of substitutions per site. Red diamonds indicate Iceland isolates. (PDF) [file pone.0092075.s007.pdf]

# HA H3

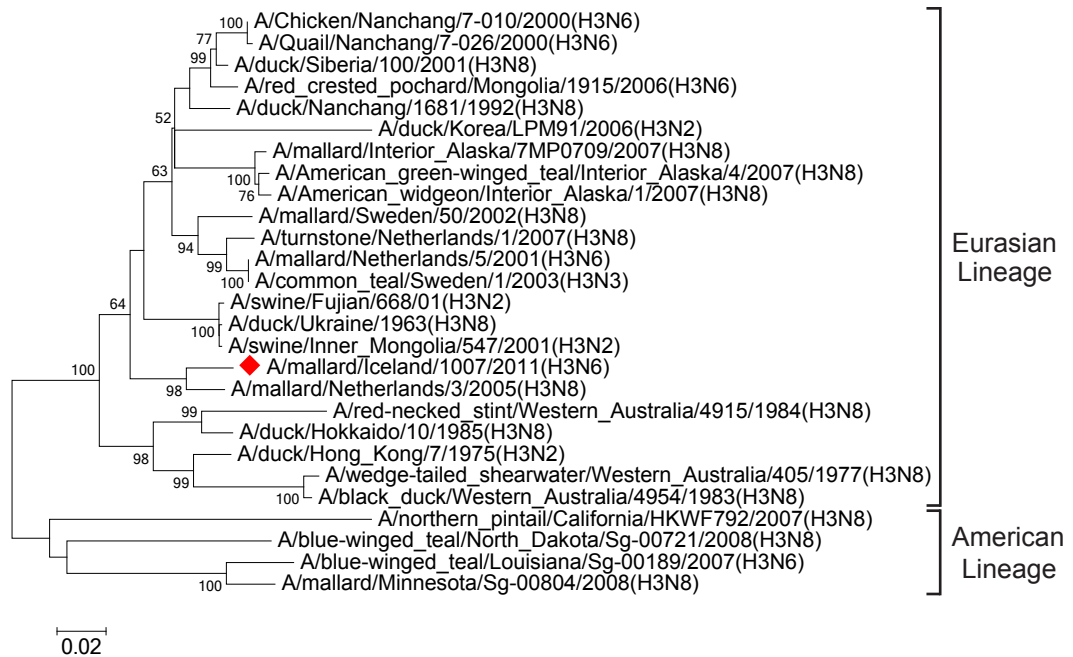

Supplement: Figure S8 — Maximum Likelihood analysis of avian influenza virus segment HA H3. Evolutionary history was inferred using the Maximum Likelihood analysis using the Tamura-Nei substitution model in Mega 5.05. A total of 1000 bootstrap replicates were used. Percentages of replicate trees (when ≥50%) in which the associated taxa clustered together are shown next to the branches. The tree is drawn to scale, with branch lengths measured in the number of substitutions per site. Red diamonds indicate Iceland isolates. (PDF) [file pone.0092075.s008.pdf]

# HA H4

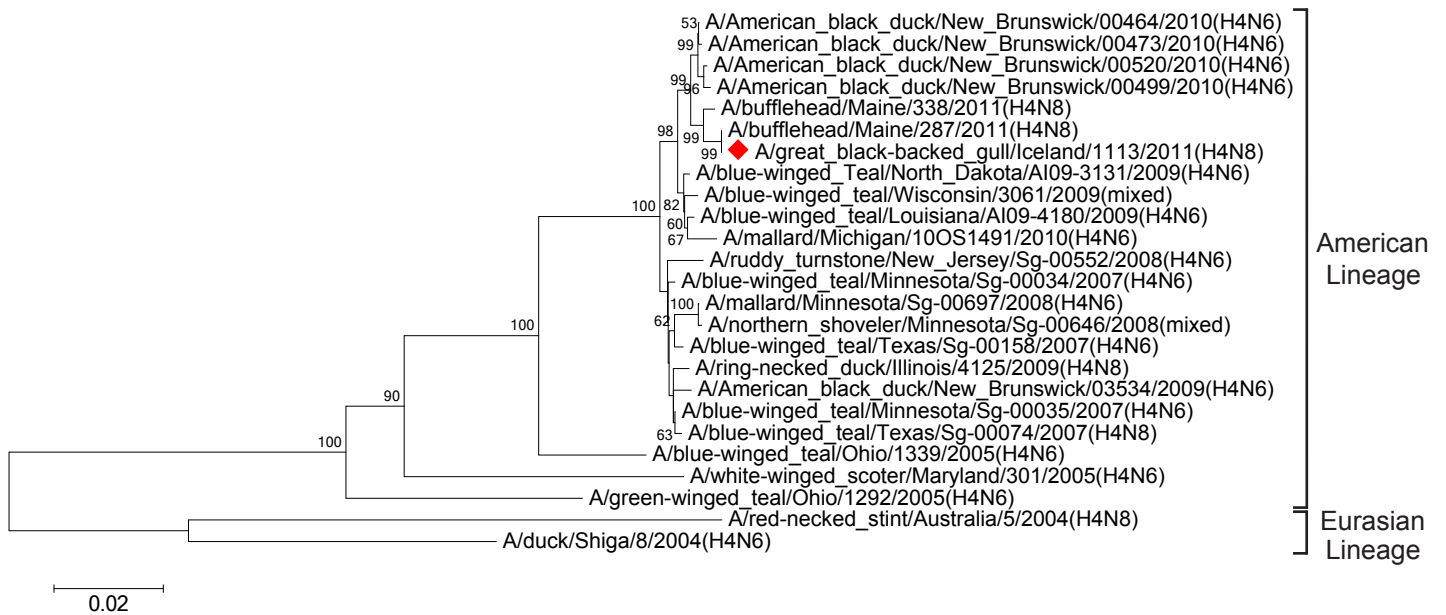

Supplement: Figure S9 — Maximum Likelihood analysis of avian influenza virus segment HA H4. Evolutionary history was inferred using the Maximum Likelihood analysis using the Tamura-Nei substitution model in Mega 5.05. A total of 1000 bootstrap replicates were used. Percentages of replicate trees (when ≥50%) in which the associated taxa clustered together are shown next to the branches. The tree is drawn to scale, with branch lengths measured in the number of substitutions per site. Red diamonds indicate Iceland isolates. (PDF) [file pone.0092075.s009.pdf]

## HA H5

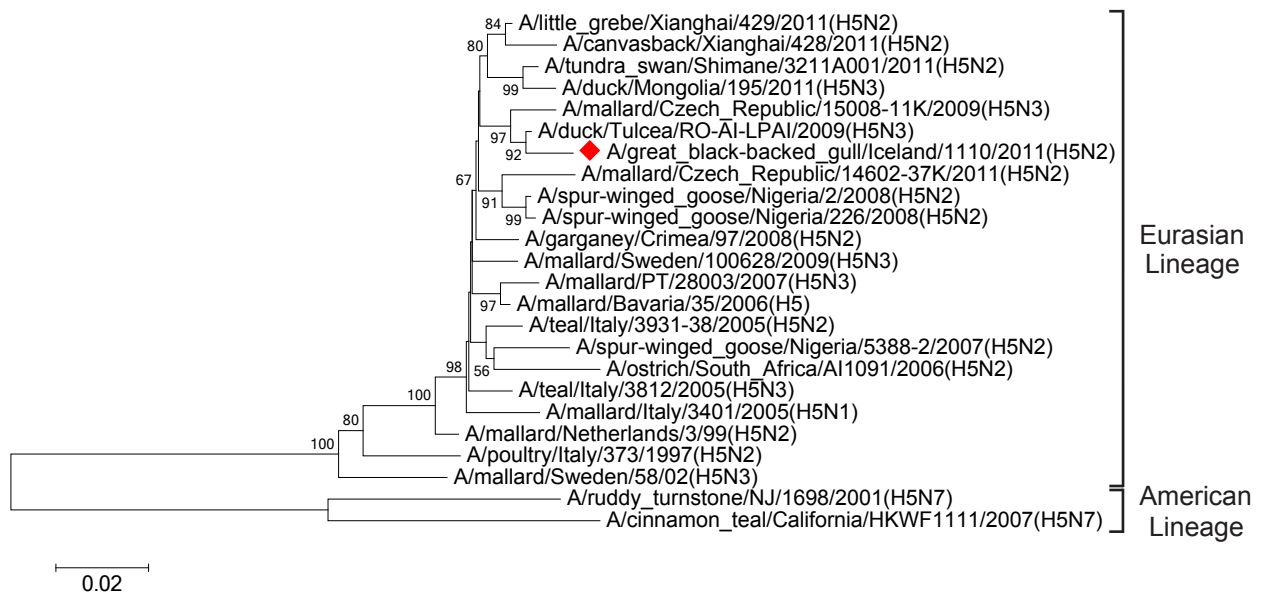

Supplement: Figure S10 — Maximum Likelihood analysis of avian influenza virus segment HA H5. Evolutionary history was inferred using the Maximum Likelihood analysis using the Tamura-Nei substitution model in Mega 5.05. A total of 1000 bootstrap replicates were used. Percentages of replicate trees (when ≥50%) in which the associated taxa clustered together are shown next to the branches. The tree is drawn to scale, with branch lengths measured in the number of substitutions per site. Red diamonds indicate Iceland isolates. (PDF) [file pone.0092075.s010.pdf]

# HA H6

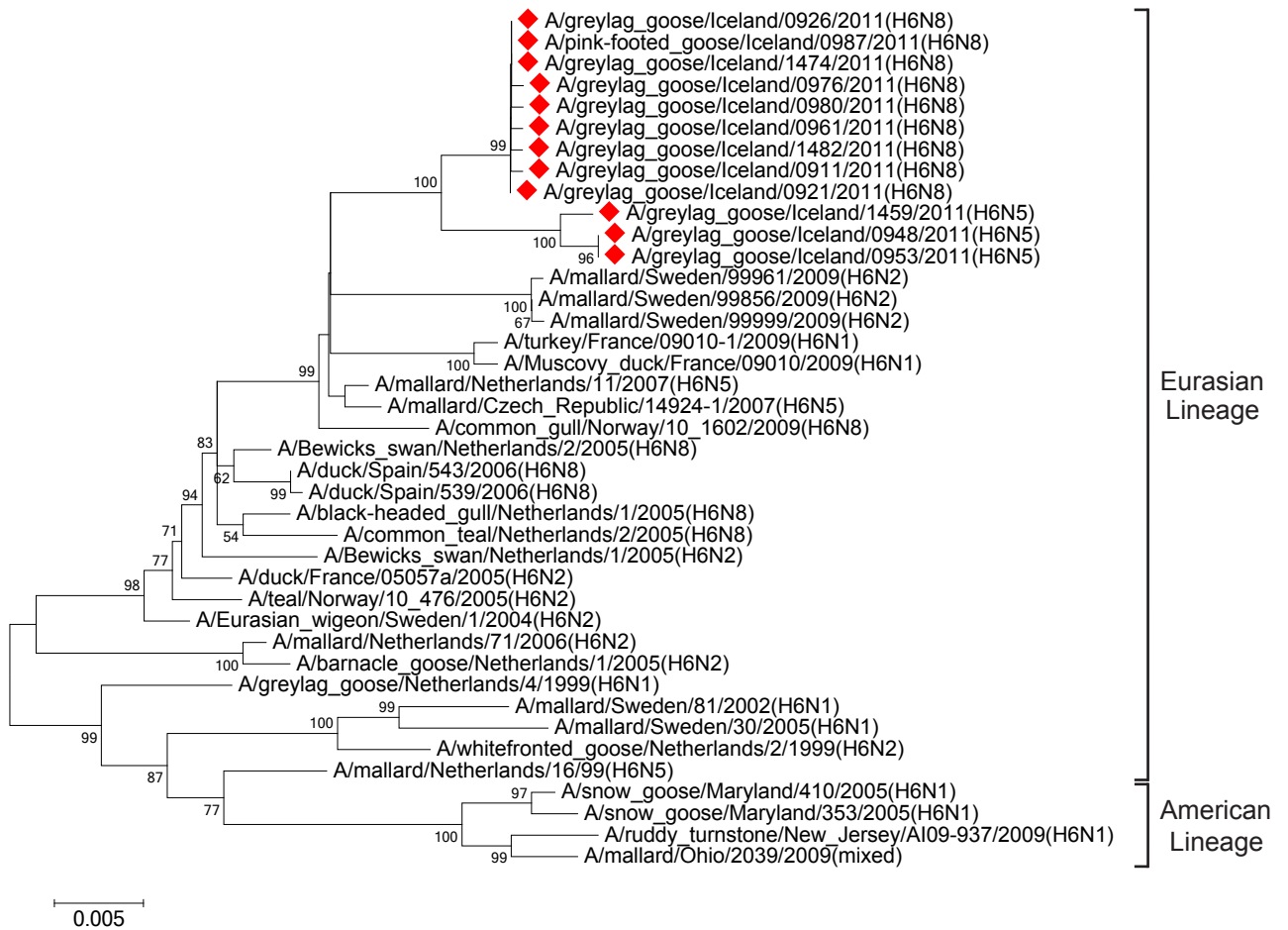

Supplement: Figure S11 — Maximum Likelihood analysis of avian influenza virus segment HA H6. Evolutionary history was inferred using the Maximum Likelihood analysis using the Tamura-Nei substitution model in Mega 5.05. A total of 1000 bootstrap replicates were used. Percentages of replicate trees (when ≥50%) in which the associated taxa clustered together are shown next to the branches. The tree is drawn to scale, with branch lengths measured in the number of substitutions per site. Red diamonds indicate Iceland isolates. (PDF) [file pone.0092075.s011.pdf]

# HA H10

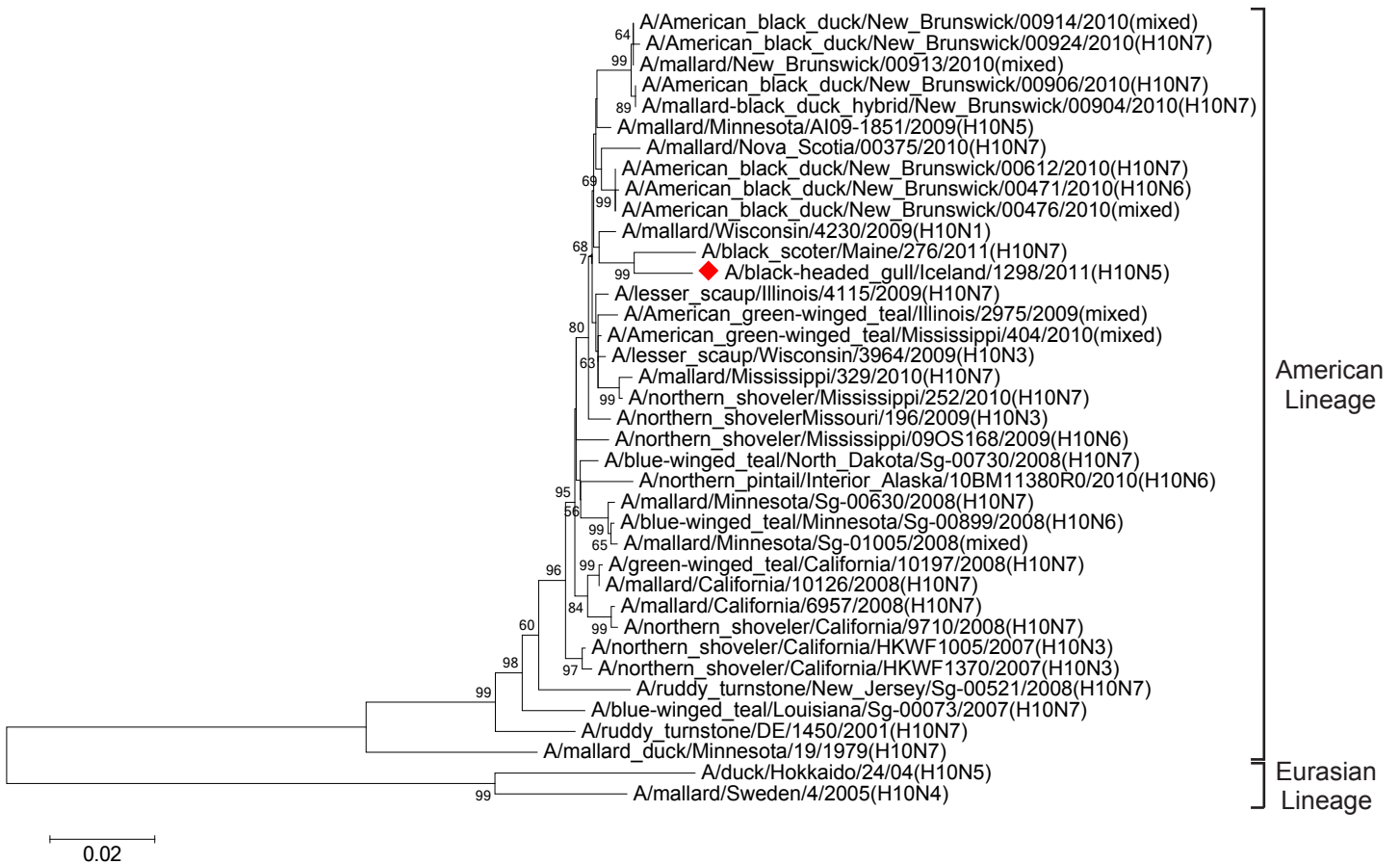

Supplement: Figure S12 — Maximum Likelihood analysis of avian influenza virus segment HA H10. Evolutionary history was inferred using the Maximum Likelihood analysis using the Tamura-Nei substitution model in Mega 5.05. A total of 1000 bootstrap replicates were used. Percentages of replicate trees (when ≥50%) in which the associated taxa clustered together are shown next to the branches. The tree is drawn to scale, with branch lengths measured in the number of substitutions per site. Red diamonds indicate Iceland isolates. (PDF) [file pone.0092075.s012.pdf]

# HA H11

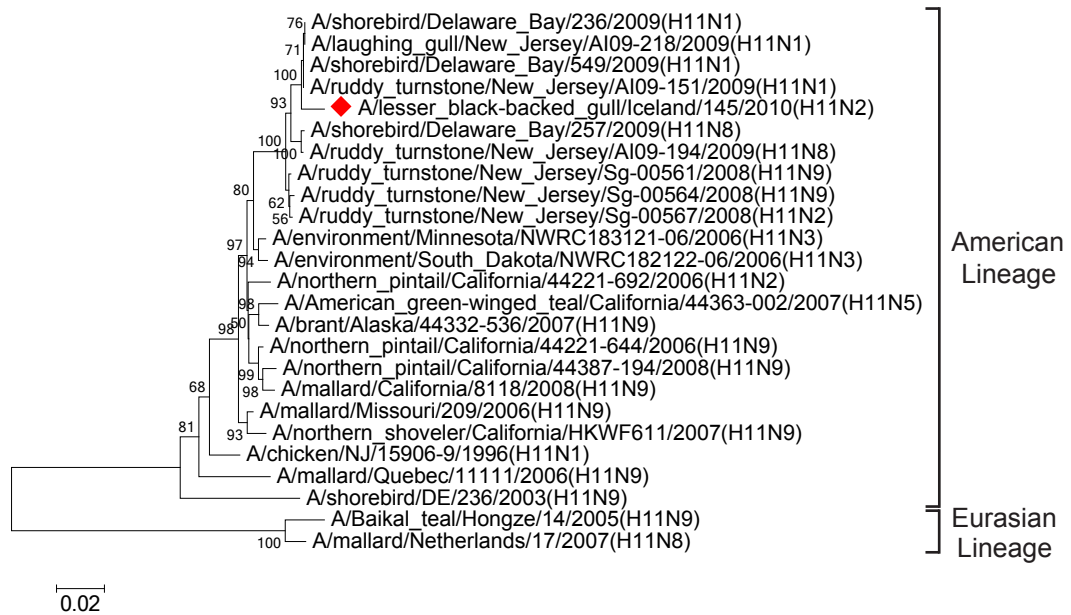

Supplement: Figure S13 — Maximum Likelihood analysis of avian influenza virus segment HA H11. Evolutionary history was inferred using the Maximum Likelihood analysis using the Tamura-Nei substitution model in Mega 5.05. A total of 1000 bootstrap replicates were used. Percentages of replicate trees (when ≥50%) in which the associated taxa clustered together are shown next to the branches. The tree is drawn to scale, with branch lengths measured in the number of substitutions per site. Red diamonds indicate Iceland isolates. (PDF) [file pone.0092075.s013.pdf]

# HA H16

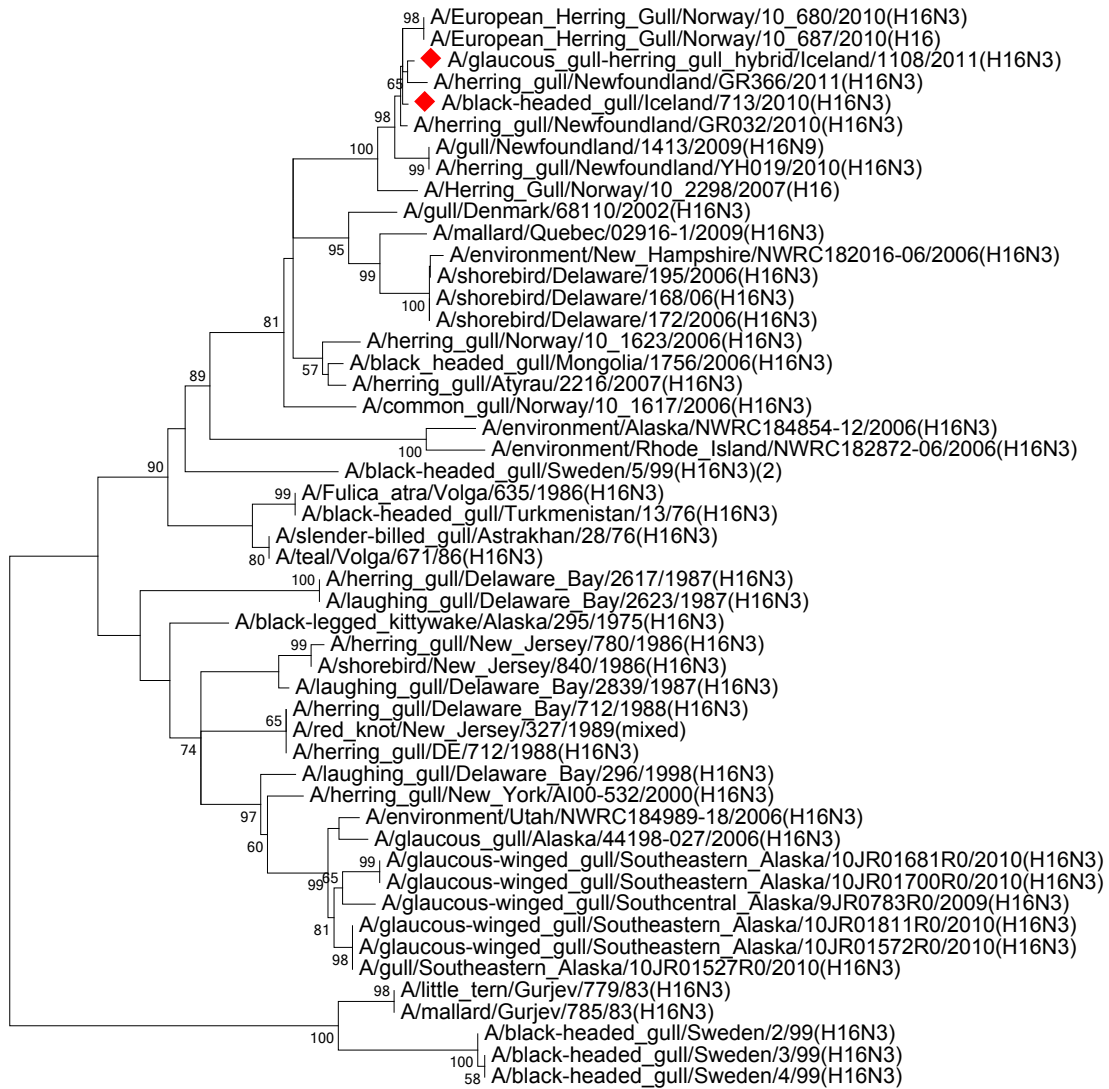

0.02

Supplement: Figure S14 — Maximum Likelihood analysis of avian influenza virus segment HA H16. Evolutionary history was inferred using the Maximum Likelihood analysis using the Tamura-Nei substitution model in Mega 5.05. A total of 1000 bootstrap replicates were used. Percentages of replicate trees (when ≥50%) in which the associated taxa clustered together are shown next to the branches. The tree is drawn to scale, with branch lengths measured in the number of substitutions per site. Red diamonds indicate Iceland isolates. (PDF) [file pone.0092075.s014.pdf]

# NA N2

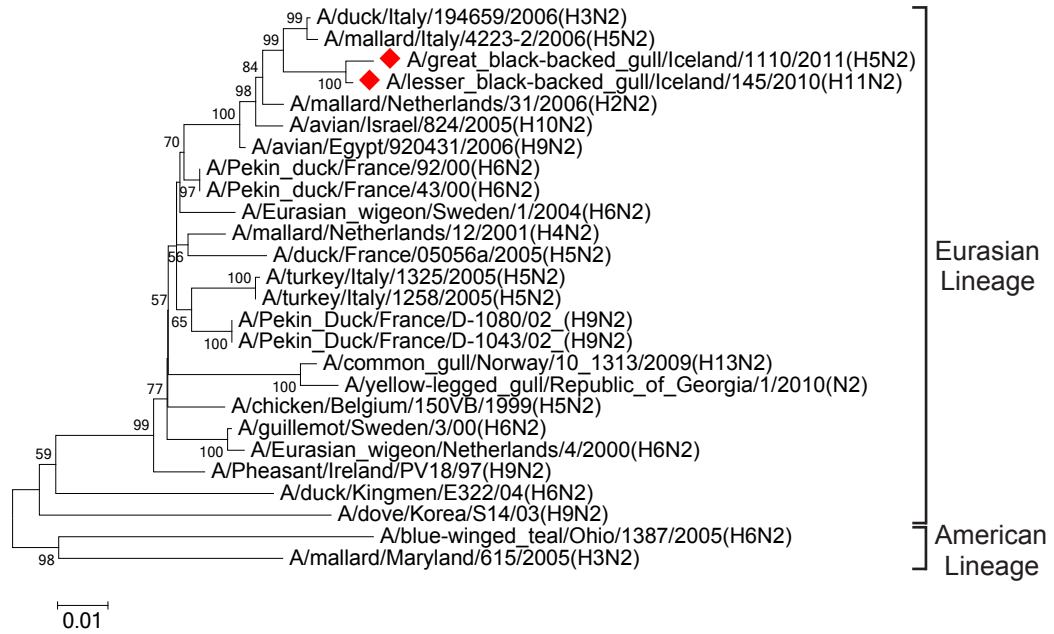

Supplement: Figure S15 — Maximum Likelihood analysis of avian influenza virus segment NA N2. Evolutionary history was inferred using the Maximum Likelihood analysis using the Tamura-Nei substitution model in Mega 5.05. A total of 1000 bootstrap replicates were used. Percentages of replicate trees (when ≥50%) in which the associated taxa clustered together are shown next to the branches. The tree is drawn to scale, with branch lengths measured in the number of substitutions per site. Red diamonds indicate Iceland isolates. (PDF) [file pone.0092075.s015.pdf]

# NA N3

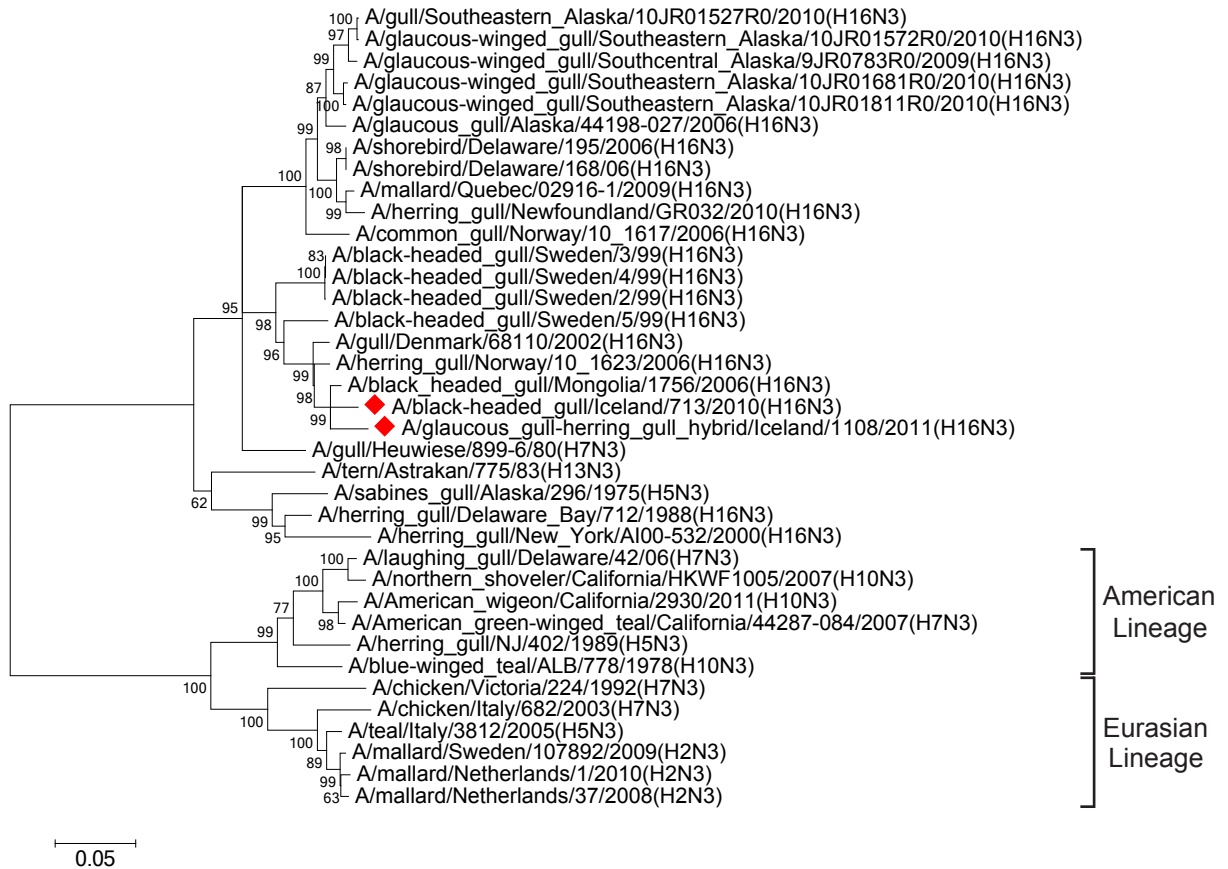

Supplement: Figure S16 — Maximum Likelihood analysis of avian influenza virus segment NA N3. Evolutionary history was inferred using the Maximum Likelihood analysis using the Tamura-Nei substitution model in Mega 5.05. A total of 1000 bootstrap replicates were used. Percentages of replicate trees (when ≥50%) in which the associated taxa clustered together are shown next to the branches. The tree is drawn to scale, with branch lengths measured in the number of substitutions per site. Red diamonds indicate Iceland isolates. (PDF) [file pone.0092075.s016.pdf]

# NA N5

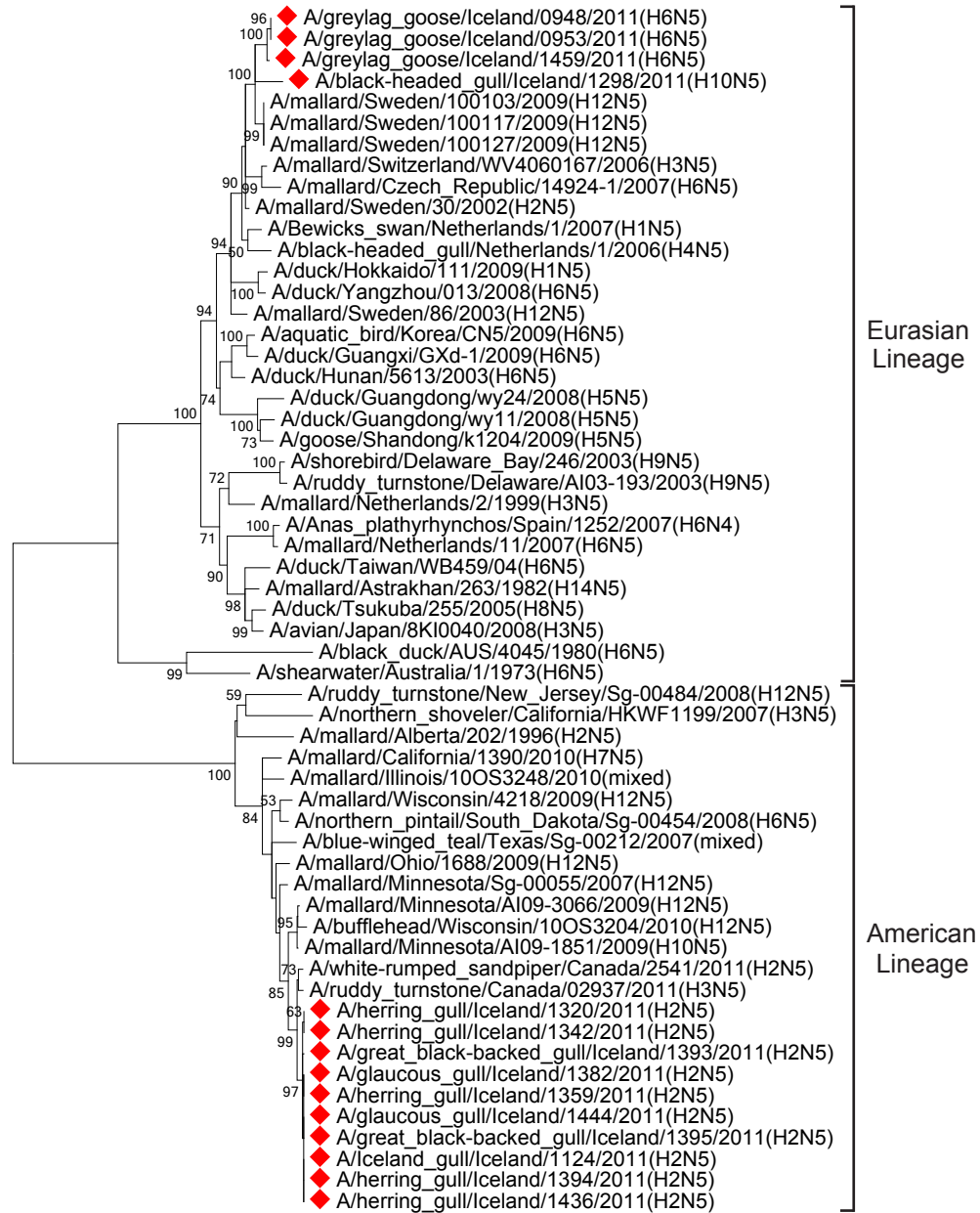

Supplement: Figure S17 — Maximum Likelihood analysis of avian influenza virus segment NA N5. Evolutionary history was inferred using the Maximum Likelihood analysis using the Tamura-Nei substitution model in Mega 5.05. A total of 1000 bootstrap replicates were used. Percentages of replicate trees (when ≥50%) in which the associated taxa clustered together are shown next to the branches. The tree is drawn to scale, with branch lengths measured in the number of substitutions per site. Red diamonds indicate Iceland isolates. (PDF) [file pone.0092075.s017.pdf]

## NA N6

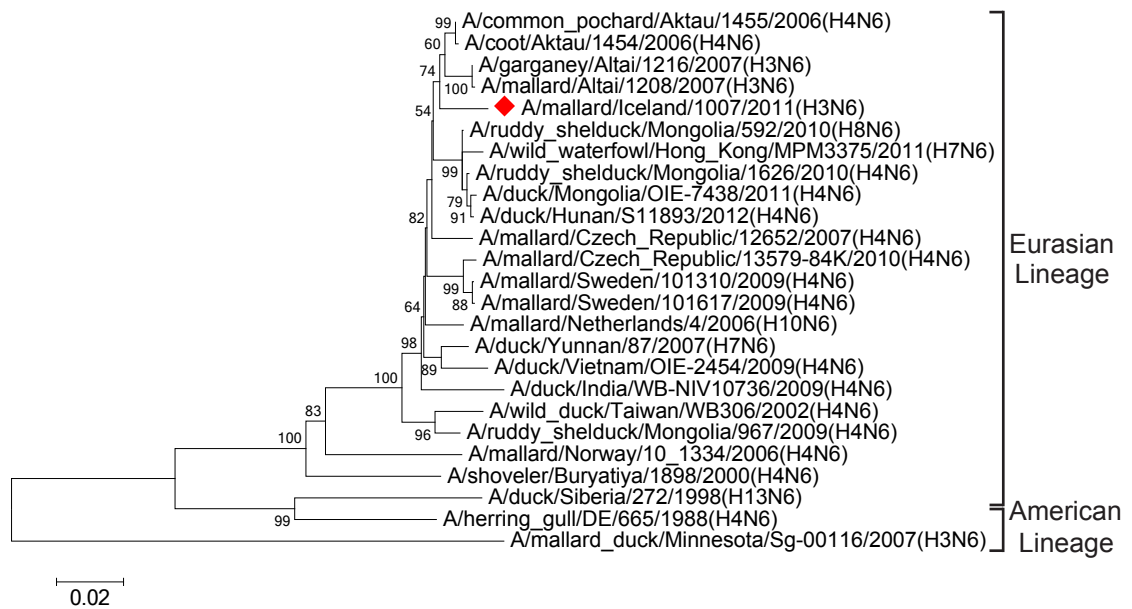

Supplement: Figure S18 — Maximum Likelihood analysis of avian influenza virus segment NA N6. Evolutionary history was inferred using the Maximum Likelihood analysis using the Tamura-Nei substitution model in Mega 5.05. A total of 1000 bootstrap replicates were used. Percentages of replicate trees (when ≥50%) in which the associated taxa clustered together are shown next to the branches. The tree is drawn to scale, with branch lengths measured in the number of substitutions per site. Red diamonds indicate Iceland isolates. (PDF) [file pone.0092075.s018.pdf]

# NA N8

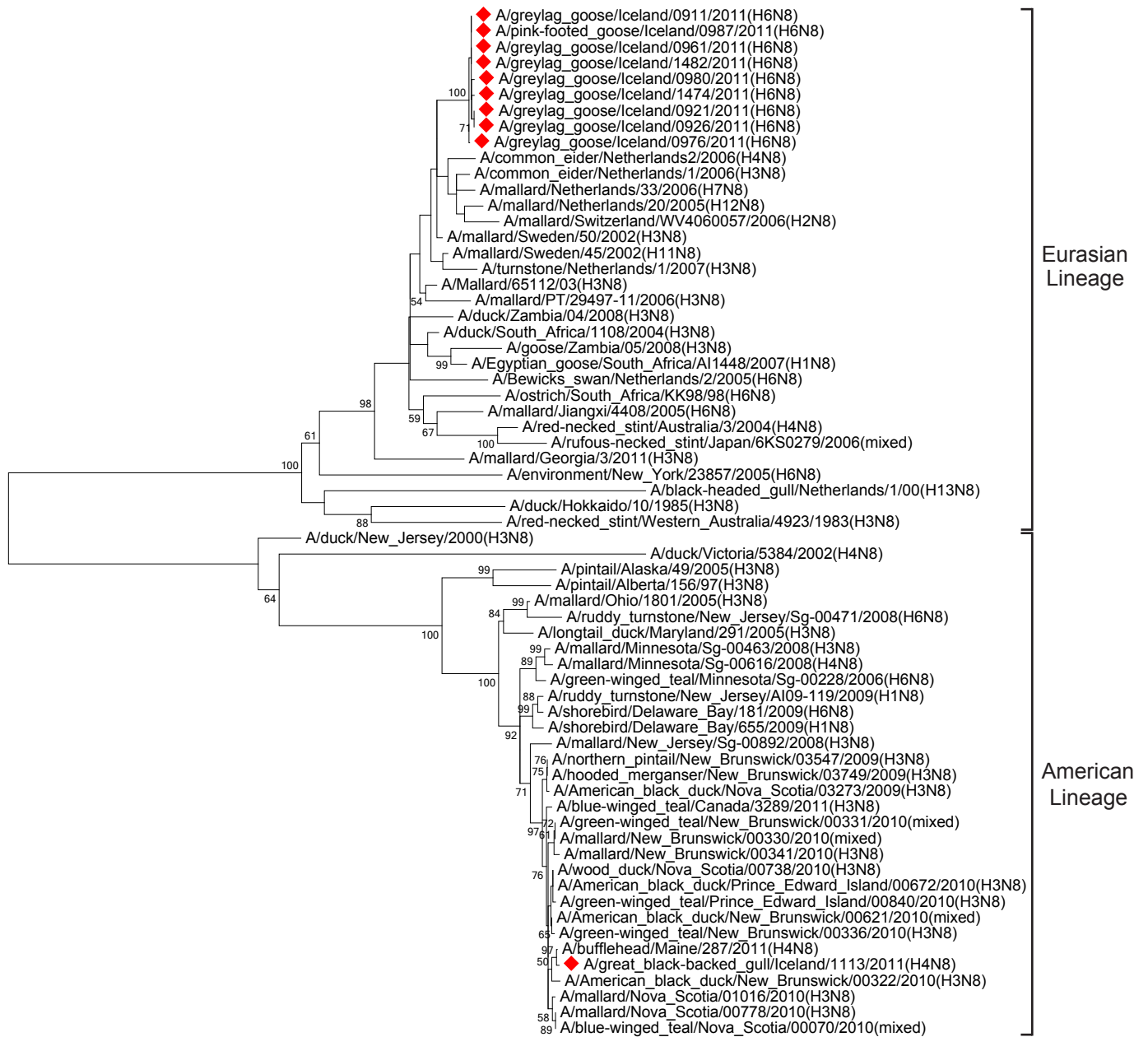

Supplement: Figure S19 — Maximum Likelihood analysis of avian influenza virus segment NA N8. Evolutionary history was inferred using the Maximum Likelihood analysis using the Tamura-Nei substitution model in Mega 5.05. A total of 1000 bootstrap replicates were used. Percentages of replicate trees (when ≥50%) in which the associated taxa clustered together are shown next to the branches. The tree is drawn to scale, with branch lengths measured in the number of substitutions per site. Red diamonds indicate Iceland isolates. (PDF) [file pone.0092075.s019.pdf]
