# Supplementary material for: North Atlantic Migratory Bird Flyways Provide Routes for Intercontinental Movement of Avian Influenza Viruses
Source: PLoS One. 2014 Mar 19;9(3):e92075. doi: 10.1371/journal.pone.0092075 (PMC3960164; doi:10.1371/journal.pone.0092075)
Supplement: Table S1 — Total cloacal swabs obtained and total virus positive for avian influenza virus from wild birds in Iceland, 2010–2011. Apparent prevalence for those species with viruses isolated shown in parenthesis. (DOCX) [file pone.0092075.s020.docx]

Table S1. Total cloacal swabs obtained and total virus positive for avian influenza virus from wild birds in Iceland, 2010-2011. Apparent prevalence for those species with viruses isolated shown in parenthesis.

| Species | Total | No. Positive |
| --- | --- | --- |
| Northern Gannet *Morus bassanus* | 1 | 0 |
| Great Cormorant *Phalacrocorax carbo* | 2 | 0 |
| European Shag *Phalacrocorax aristotelis* | 6 | 0 |
| Pink-footed Goose *Anser brachyrhynchus* | 13 | 1 (7.7%) |
| Greylag Goose *Anser anser* | 223 | 11 (4.9%) |
| Mallard *Anas platyrhynchos* | 14 | 1 (7.1%) |
| Common Eider *Somateria mollissima* | 35 | 0 |
| Eurasian Oystercatcher *Haematopus ostralegus* | 16 | 0 |
| Common Ringed Plover *Charadrius hiaticula* | 4 | 0 |
| Common Snipe *Gallinago gallinago* | 1 | 0 |
| Common Redshank *Tringa totanus* | 1 | 0 |
| Ruddy Turnstone *Arenaria interpres* | 64 | 0 |
| Red Knot *Calidris canutus* | 1 | 0 |
| Sanderling *Calidris alba* | 129 | 0 |
| Dunlin *Calidris alpina* | 2 | 0 |
| Purple Sandpiper *Calidris maritima* | 2 | 0 |
| Common Gull *Larus canus* | 1 | 0 |
| Great Black-backed Gull *Larus marinus* | 38 | 4 (10.5%) |
| Glaucous Gull *Larus hyperboreus* | 98 | 2 (2.0%) |
| Iceland Gull *Larus glaucoides* | 19 | 1 (5.3%) |
| Herring Gull *Larus argentatus* | 121 | 5 (4.1%) |
| Glaucous Gull x Herring Gull hybrid *Larus hyperboreus x L. argentatus* | 12 | 1 (8.3%) |
| Lesser Black-backed Gull *Larus fuscus* | 96 | 1 (1.0%) |
| Black-headed Gull *Chroicocephalus ridibundus* | 168 | 2 (1.2%) |
| Common Murre *Uria aalge* | 1 | 0 |
| Black Guillimot *Cepphus grylle* | 10 | 0 |
| Total | 1078 | 29 (2.7%) |
